# Management of Synovial Chondromatosis of the Elbow With Ulnar Nerve Palsy by Open Approach: A Case Report

**DOI:** 10.7759/cureus.59807

**Published:** 2024-05-07

**Authors:** Mainak Roy, Deepanjan Das, Pratik Shahare, Samir Dwidmuthe, Denish Chandrakar

**Affiliations:** 1 Orthopaedics, All India Institute of Medical Sciences, Nagpur, Nagpur, IND

**Keywords:** surgical management, elbow joint, open approach surgery, ulnar nerve palsy, synovial chondromatosis

## Abstract

Synovial chondromatosis is a rare condition characterized by benign metaplasia of the synovial membrane, leading to nodular growths within the joint space.

We present the case of a 58-year-old woman with persistent pain and stiffness in her right elbow, ultimately diagnosed with synovial chondromatosis. Examination revealed joint effusion, tenderness, and restricted range of motion, with palpable loose bodies and ulnar nerve symptomatology. X-ray confirmed the diagnosis. Open synovectomy was performed, with meticulous attention to ulnar nerve protection and decompression. Postoperative care included analgesics, anti-inflammatories, and physiotherapy.

Synovial chondromatosis of the elbow requires prompt diagnosis and surgical intervention to alleviate symptoms and prevent complications. Prognosis is favorable with complete removal of the affected tissue. Ulnar nerve palsy should be carefully addressed during surgical management.

## Introduction

Primary synovial chondromatosis is a rare, benign condition where the joint synovium undergoes benign metaplasia. This growth leads to the formation of nodules, consisting of chondrocytes, within the synovial membrane [1]. Sometimes, these nodules may detach and become loose bodies within the joint space [2]. These loose bodies can ossify, a condition called synovial osteochondromatosis [2]. Recent studies suggest that genetic abnormalities (clonal karyotypic abnormalities of chromosome 6) may play a role in its development, suggesting a neoplastic etiology [3]. While it can affect any joint, it's most commonly seen in the knee and elbow joints. The elbow joint is particularly prone to this condition due to repetitive stress [4]. Three stages have been described by Milgram (in 1977): phase I, active inflammation without loose bodies; phase II, nodular inflammation with loose bodies; and phase III, presence of loose bodies with reduced inflammation [5]. Symptoms can vary, including inflammation, mechanical symptoms, such as restricted range of motion, and joint locking as well as pain. Pressure on the adjacent nerves leads not only to pain but also to nerve dysfunction [4]. Diagnosis can be delayed as symptoms are often characterized by incision onset [5]. Other conditions that may mimic synovial chondromatosis should be considered during diagnosis. These include secondary synovial chondromatosis, which arises from inflammation and loose body formation in the synovial membrane due to conditions like osteoarthritis, trauma, or osteochondritis dissecans. Additionally, differential diagnoses should encompass more serious conditions such as synovial chondrosarcoma, calcifying aponeurotic fibroma, hydroxyapatite deposition, pigmented villonodular synovitis, elbow tuberculosis, and rheumatoid arthritis [1]. Pain relief in the initial phase of synovial chondromatosis can often be managed non-operatively using nonsteroidal anti-inflammatory drugs (NSAIDs) and corticosteroid injections. However, for phases II and III, surgical treatment is usually recommended as the primary option by most experts. This typically involves either arthroscopic or open surgery to remove the abnormal tissue (synovectomy) and any loose bodies within the joint [1]. While both approaches can be effective, arthroscopic surgery offers several advantages such as a quicker recovery time and higher patient satisfaction [6].

## Case presentation

**Figure 1:**
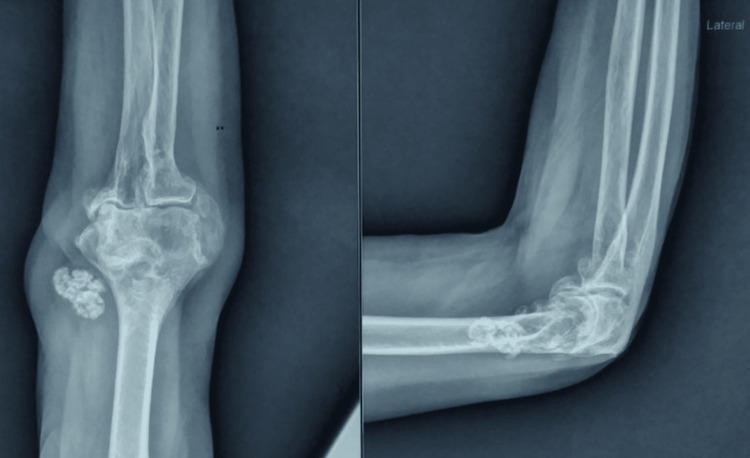
Preoperative X-ray (anteroposterior and lateral views) showing multiple loose bodies in the right elbow

A 58-year-old female patient was referred to our clinics because of persistent pain and stiffness in her right elbow over the past year. The pain was diffuse, insidious in onset, and gradually progressive and aggravated with movement. Previous elbow surgery or trauma was not reported, and she was otherwise healthy. During the examination, there was noticeable swelling around her right elbow joint, and tenderness was evident along the medial joint line. Upon palpation, multiple loose bodies were detected on the posterior and medial aspects of her right elbow. Additionally, her elbow range of motion was restricted to 30-110 degrees. Clawing of little and ring fingers of the affected side was also observed, indicating potential ulnar nerve involvement. Further, there was a decrease in the strength of the intrinsic muscles of her hand, suggestive of ulnar nerve impairment. An X-ray of her right elbow (Figure 1) revealed synovial chondromatosis, a condition characterized by the presence of loose bodies within the joint. Electromyography and nerve conduction studies were done and suggestive of compression of the ulnar nerve at the elbow joint with reduced conduction velocity. Magnetic resonance imaging (MRI) of the ipsilateral elbow joint suggested multiple T1/T2 hypointense lesions showing blooming on gradient recalled echo (GRE) just above the medial epicondyle of the humerus and the possibility of synovial chondromatosis.

**Figure 2:**
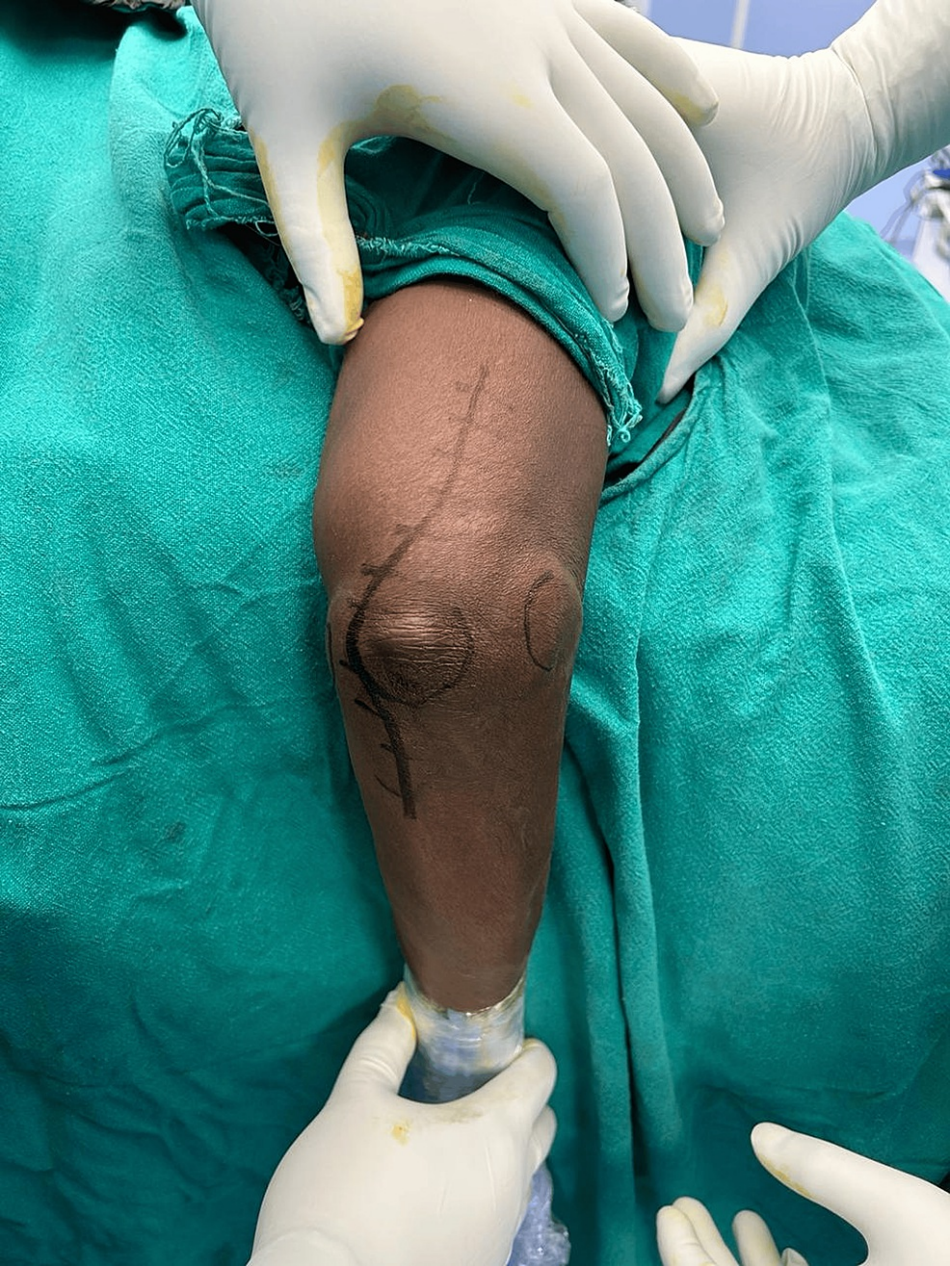
The skin incision was planned with a slight medial curve to facilitate ulnar nerve exploration

Consequently, she was scheduled for open synovectomy, a surgical procedure aimed at removing the abnormal synovial tissue and loose bodies along with ulnar nerve neurolysis and decompression. The surgery was performed in the lateral decubitus position under a tourniquet with pressure set to 250 mm Hg, through a dorsal midline incision with a slight medial curve to explore the ulnar nerve (Figure 2).

**Figure 3:**
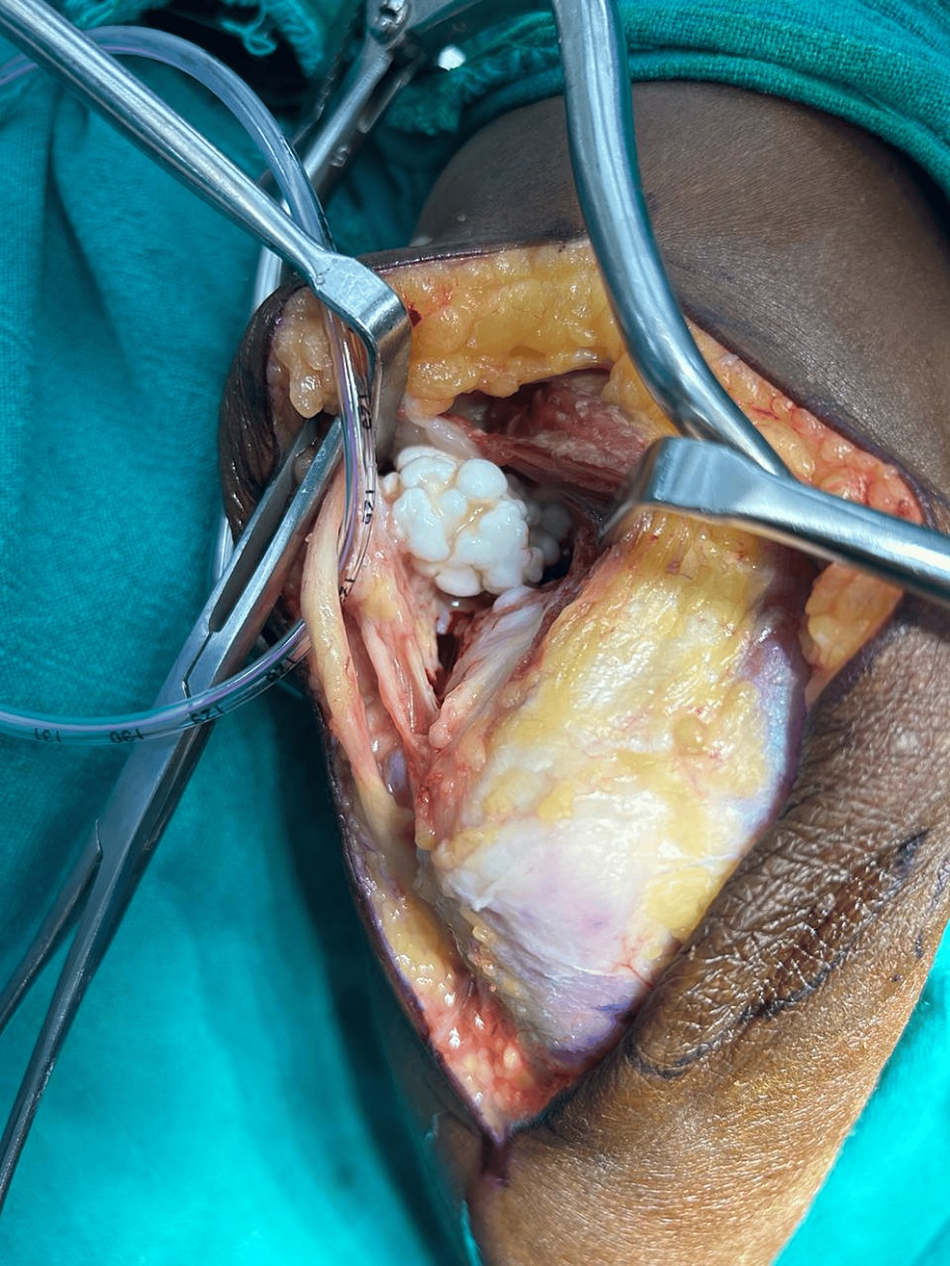
Intraoperative image: after dissection of the underlying subcutaneous tissue and fascia, the ulnar nerve was secured and loose body visualized

**Figure 4:**
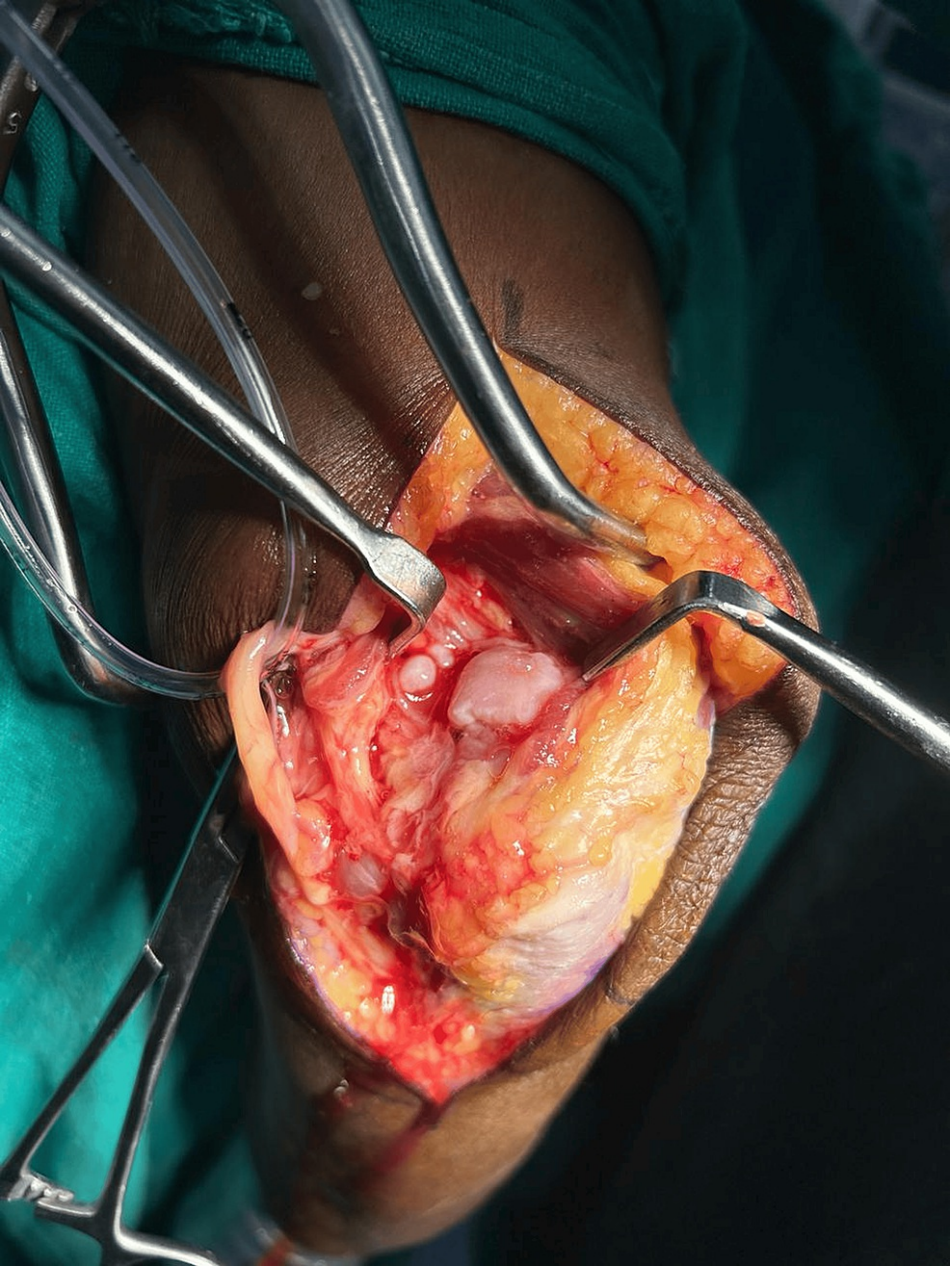
Removal of loose bodies adjacent to the triceps

Careful dissection was conducted to access the underlying tissues, ensuring the protection of the ulnar nerve (Figure 3 and Figure 4).

**Figure 5:**
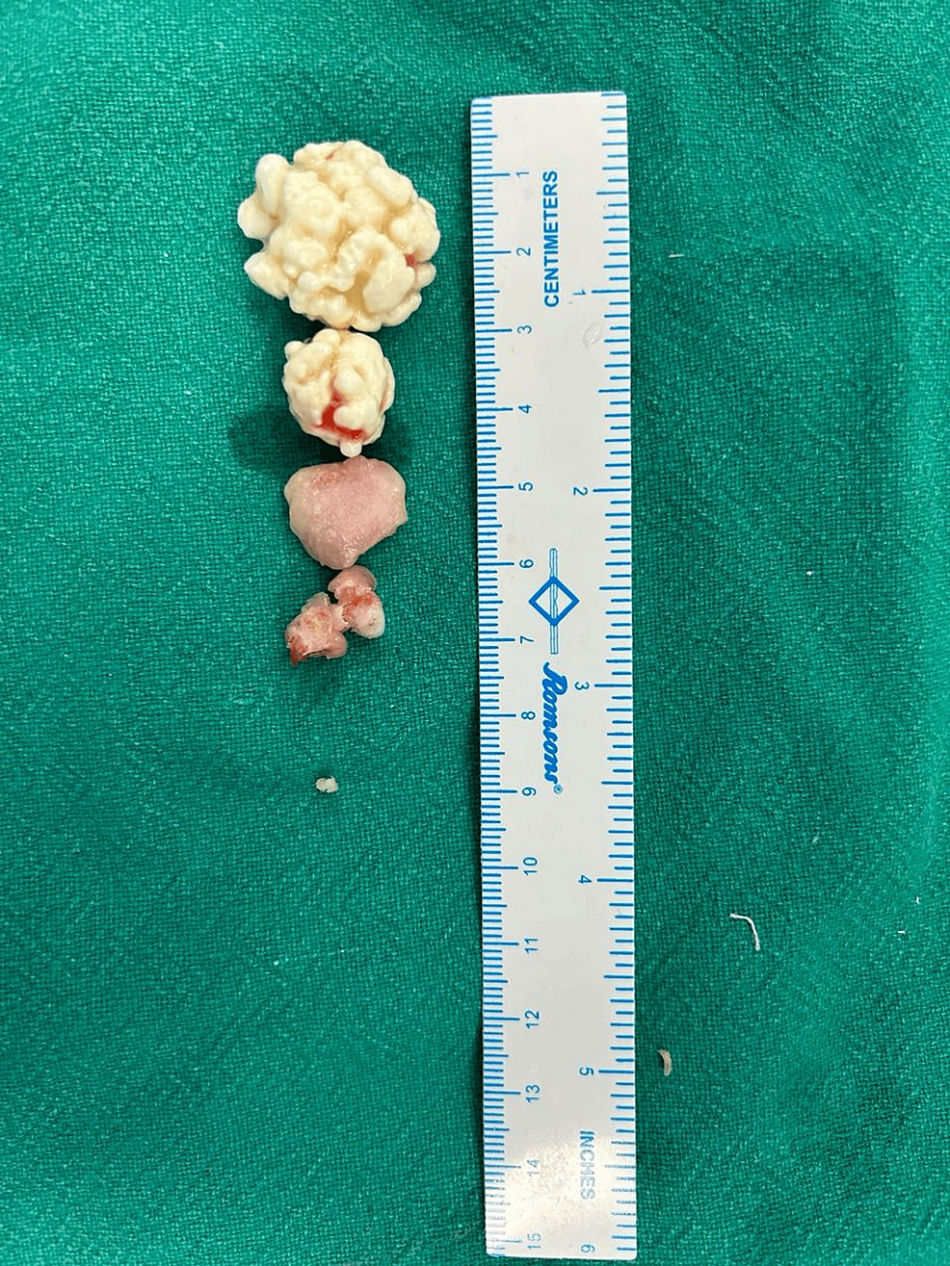
Intraoperative image showing all removed multi-lobulated loose bodies from the elbow joint

Subsequently, the thickened synovium containing loose bodies was excised, and exploration and decompression of the ulnar nerve were performed (Figure 5).

**Figure 6:**
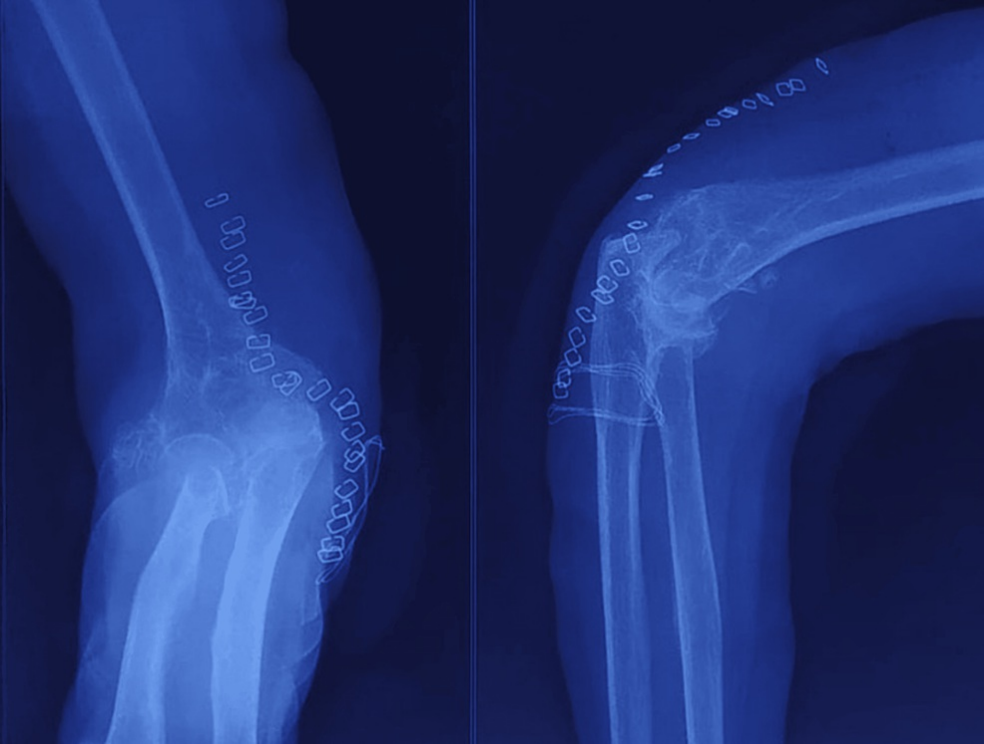
Postoperative X-ray

Postoperative X-ray was performed to assess the removal of the majority of the loose bodies from the elbow joint (Figure 6).

**Figure 7:**
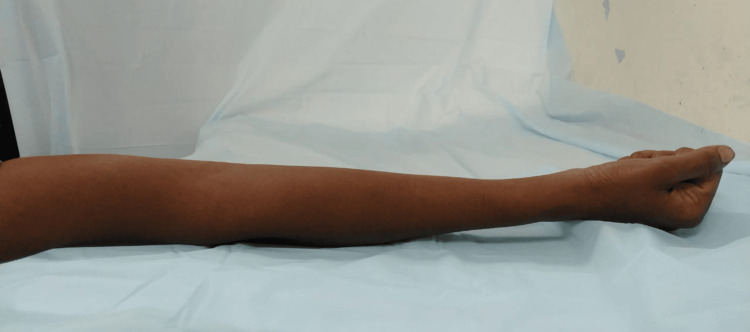
One-year postoperative follow-up image showing complete extension of the elbow

**Figure 8:**
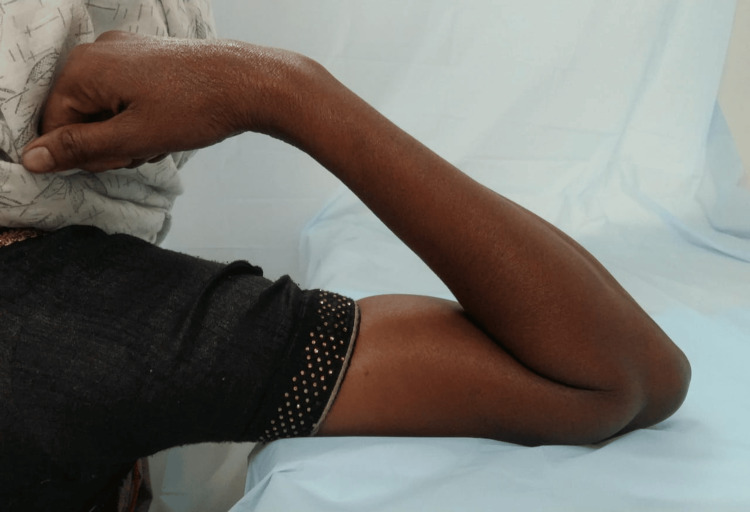
One-year postoperative follow-up image showing complete flexion of the elbow

The patient received analgesics (indomethacin 75 mg twice daily for five days) to reduce postoperative pain and was instructed to start immediate postoperative physiotherapy sessions. Regular follow-up was also advised. Significant clinical improvement was evident at the six-week follow-up with a postoperative range of motion of 0-130 (eg) degrees. Gradual resolution of pain, weakness, and clawing were also noted. Post-one-year follow-up, the patient's elbow range of motion improved significantly with no residual weakness and clawing in her hand (Figure 7 and Figure 8). She was able to carry out her daily activities effectively.

## Discussion

Synovial chondromatosis is a benign proliferative condition affecting the synovium, typically found in the synovium of joints, resulting in the development of multiple cartilaginous nodules or loose bodies [4]. It commonly affects individuals aged 30-50, with a male-to-female ratio of around 1.8 to 1, but is rare in children and adolescents [7]. This condition usually affects large joints such as the knee and the hip but can occur in any joint, including the temporomandibular joints, hips, elbows, ankles, and shoulders, and has even been reported in the spinal cord [4,8]. It can manifest as pain, swelling, limited motion, palpable masses, and joint locking [5]. Diagnosis can be challenging due to its nonspecific symptoms, and it may be mistaken for other conditions like synovial chondrosarcoma, pigmented villonodular synovitis, or rheumatoid arthritis [9]. Imaging techniques such as X-rays, CT scans, and MRI are essential for diagnosis, with MRI being particularly useful for assessing the extent of the lesion and involvement of surrounding tissues [4]. Treatment typically involves the surgical removal of loose bodies and synovectomy, particularly in the early stages, to prevent secondary osteoarthritis. Arthroscopic surgery is often preferred due to its advantages in visualization, safety, and quicker recovery, although the choice between open and arthroscopic methods depends on individual cases [4]. Recurrence of synovial chondromatosis after surgical treatment can occur in up to 22% of cases, often due to the incomplete removal of the affected synovium or loose bodies [4]. Complete removal of all involved synovial tissue is recommended to reduce the risk of recurrence and potential malignant transformation into chondrosarcoma [1]. In our case, due to the necessity of removing potential extraarticular loose bodies, we opted for the open surgical approach. This decision was made to ensure better visibility and increase the likelihood of completely removing the affected tissue, thus reducing the chances of recurrence. Moreover, the open approach was necessary in order to explore the ulnar nerve and carry out a safe and adequate decompression. Postoperatively, the patient's clinical and functional outcomes were excellent. The patient was followed up regularly over one year without any clinical or radiological signs of recurrence.

## Conclusions

Synovial chondromatosis of the elbow is a rare benign condition characterized by multiple nodules of hyaline cartilage within the connective tissue around the joint. Symptoms include pain, swelling, and restricted motion. Treatment involves removing the affected synovium and cartilage nodules to alleviate symptoms and improve joint function. Prognosis is generally favorable with a low recurrence rate if complete removal is achieved; otherwise, there may be a high failure rate and poor patient compliance. Ulnar nerve palsy could arise as a complication of compression/entrapment. However, careful dissection and proper securing of the nerve can prevent such consequences.
